# Synthesis of fluorescent analogs of relaxin family peptides and their preliminary *in vitro* and *in vivo* characterization

**DOI:** 10.3389/fchem.2013.00030

**Published:** 2013-12-06

**Authors:** Linda J. Chan, Craig M. Smith, Berenice E. Chua, Feng Lin, Ross A. D. Bathgate, Frances Separovic, Andrew L. Gundlach, Mohammed Akhter Hossain, John D. Wade

**Affiliations:** ^1^The Florey Institute of Neuroscience and Mental Health, The University of MelbourneVIC, Australia; ^2^School of Chemistry, The University of MelbourneVIC, Australia; ^3^Florey Department of Neuroscience and Mental Health, The University of MelbourneVIC, Australia; ^4^Department of Biochemistry and Molecular Biology, The University of MelbourneVIC, Australia

**Keywords:** relaxin, Cy5.5 fluorophore, click chemistry, RXFP1, RXFP2, brain

## Abstract

Relaxin, a heterodimeric polypeptide hormone, is a key regulator of collagen metabolism and multiple vascular control pathways in humans and rodents. Its actions are mediated via its cognate G-protein-coupled receptor, RXFP1 although it also “pharmacologically” activates RXFP2, the receptor for the related, insulin-like peptide 3 (INSL3), which has specific actions on reproduction and bone metabolism. Therefore, experimental tools to facilitate insights into the distinct biological actions of relaxin and INSL3 are required, particularly for studies of tissues containing both RXFP1 and RXFP2. Here, we chemically functionalized human (H2) relaxin, the RXFP1-selective relaxin analog H2:A(4-24)(F23A), and INSL3 to accommodate a fluorophore without marked reduction in binding or activation propensity. Chemical synthesis of the two chains for each peptide was followed by sequential regioselective formation of their three disulfide bonds. Click chemistry conjugation of Cy5.5 at the B-chain N-terminus, with conservation of the disulfide bonds, yielded analogs displaying appropriate selective binding affinity and ability to activate RXFP1 and/or RXFP2 *in vitro*. The *in vivo* biological activity of Cy5.5-H2 relaxin and Cy5.5-H2:A(4-24)(F23A) was confirmed in mice, as acute intracerebroventricular (icv) infusion of these peptides (but not Cy5.5-INSL3) stimulated water drinking, an established behavioral response elicited by central RXFP1 activation. The central distribution of Cy5.5-conjugated peptides was examined in mice killed 30 min after infusion, revealing higher fluorescence within brain tissue near-adjacent to the cerebral ventricle walls relative to deeper brain areas. Production of fluorophore-conjugated relaxin family peptides will facilitate future pharmacological studies to probe the function of H2 relaxin/RXFP1 and INSL3/RXFP2 signaling *in vivo* while tracking their distribution following central or peripheral administration.

## Introduction

Relaxin is a peptide hormone that has long been recognized for its pleiotropic roles in peripheral tissues, especially during pregnancy (Hisaw, 1926; Bathgate et al., 2013). It is a 53 amino acid peptide (6 kDa) that comprises two chains (A and B) and three disulfide bonds, one intramolecular disulfide bond within the A-chain and two others linking the A- and B-chains (Shabanpoor et al., 2009; Chan et al., 2011). In addition to its roles in reproductive physiology, relaxin (designated H2 relaxin in humans) has physiological and therapeutic vasodilatory, cardio-protective and anti-fibrotic actions in animals and human. In fact, the peptide has recently passed Phase III clinical trials for the treatment of acute heart failure (Teerlink et al., 2013).

Relaxin is mainly produced in the corpus luteum during pregnancy, but is also present in the placenta and prostate gland, in addition to a host of non-reproductive tissues including the brain (Shabanpoor et al., 2009; Bathgate et al., 2013). It interacts with relaxin family peptide receptor 1 (RXFP1), a G-protein-coupled receptor, to exert its biological effects. In experimental systems H2 relaxin also interacts with RXFP2, which is structurally similar to RXFP1 (Hsu et al., 2002), and is the native receptor for the related peptide, insulin-like peptide 3 (INSL3) (Kumagai et al., 2002) although there is no strong evidence that this interaction is physiologically significant (Bogatcheva et al., 2003; Kamat et al., 2004). Relaxin displays no cross-reactivity for RXFP3 and RXFP4, the receptors for relaxin-3 and insulin-like peptide 5 (INSL5) (Shabanpoor et al., 2009). The cardio-protective effects of H2 relaxin are known to be mediated by RXFP1 (Du et al., 2010). Relaxin and RXFP1 are present in a number of different regions of the rat (Osheroff and Phillips, 1991; Ma and Gundlach, 2007) and mouse (Piccenna et al., 2005) brain, but their role there has not been clearly identified. Several investigators have reported that intracerebroventricular (icv) infusion of H2 relaxin in rodents induces a drinking (dipsogenic) response, which is thought to be mediated via activation of RXFP1 expressed by neurons in the circumventricular organs, such as the subfornical organ (Summerlee et al., 1998; Sunn et al., 2002). However, the function of receptors within deeper brain tissue more distal to the ventricle wall is not known nor is it known to what degree these receptors are accessed following infusion of H2 relaxin via the icv route. Similarly, the INSL3 receptor, RXFP2, is expressed within regions of the rat and mouse brain both proximal and distal to the ventricular system (Sedaghat et al., 2008), but little data has been reported on their function.

In this regard, examining the behavior of rodents following infusion of receptor-selective peptide ligands into the cerebral ventricular system or local brain regions has revealed that another relaxin family peptide receptor, RXFP3, has various putative central functional roles (McGowan et al., 2007; Ma et al., 2009; Tanaka, 2010; Ryan et al., 2013). Similar experimental approaches may be equally valuable for determining the central role of H2 relaxin/RXFP1 and INSL3/RXFP2 signaling. In pursuit of this goal, our recent structure-function studies led to the development of an RXFP1-selective H2 relaxin analog, H2:A(4-24)(F23A) (Chan et al., 2012). However, interpretation of studies involving infusions of H2 relaxin, H2:A(4-24)(F23A) or INSL3 into the rodent brain is currently hampered by an inability to determine how readily these peptides penetrate and spread throughout brain tissue. Such information is ultimately important, as although injected peptides are often thought to “bathe the whole brain” when delivered icv, this assumption is usually not tested.

In studies to address this long-term goal for the relaxin peptide family, we have prepared functionally active fluorescent analogs of H2 relaxin, H2:A(4-24)(F23A) and INSL3. This required careful design and development as most available fluorophores are large, bulky molecules and may potentially mask the active site of the peptide leading to significant or complete loss of activity. Furthermore, it is important to consider the possibility of an adverse impact upon brain distribution. Using chemical peptide synthesis methods and regioselective disulfide bond formation, we assembled cyanine 5.5 (Cy5.5) fluorophore-conjugated H2 relaxin (Cy5.5-H2 relaxin), which retains high affinity for RXFP1 and RXFP2. Synthetic RXFP1- and RXFP2-specific analogs, namely Cy5.5-H2:A(4-24)(F23A) and Cy5.5-INSL3, respectively, were also prepared. Attachment of the Cy5.5 fluorophore was achieved by post-synthesis azide-alkyne Huisgen cycloaddition “click chemistry,” an effective method for labeling at a specific site within these cysteine-rich relaxin family peptide analogs (Kolb et al., 2001). The biological activity of Cy5.5-H2 relaxin and Cy5.5-H2:A(4-24)(F23A) was then confirmed by observing a dipsogenic response following icv infusion, and subsequent histological analysis of brains harvested 30 min post icv infusion revealed high concentrations of all three peptides in brain gray matter adjacent to the cerebral ventricle wall.

## Materials and methods

### Solid phase peptide synthesis

**Figure 1:**
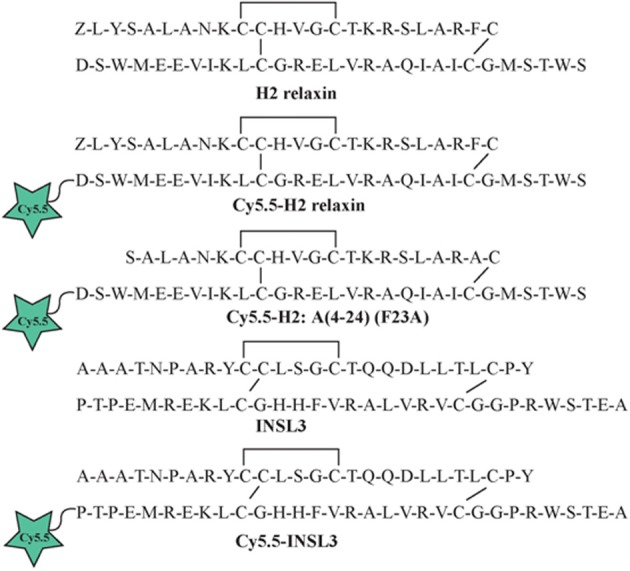
**Amino acid sequence of native H2 relaxin and INSL3 and synthetic Cy5.5 analogs**.

Individual A- and B-chains of H2 relaxin, H2:A(4-24)(F23A) and INSL3 with appropriate regioselective S-protection were synthesized using either continuous flow or microwave-assisted solid phase methodologies on an automated PerSeptives Biosystems Pioneer peptide synthesizer and a CEM Liberty peptide synthesizer, respectively (Hossain et al., 2008b, 2009). Upon complete coupling of the final amino acid of the native peptide sequence, an extra amino acid containing the alkyne group (Fmoc-L-propargylglycine) was attached using manual coupling procedures. Following simultaneous cleavage, side chain deprotection and purification of crude A- and B-chains, stepwise formation of the three disulfide bonds was conducted via oxidation, thiolysis and iodolysis consecutively (Bathgate et al., 2006; Hossain et al., 2006, 2008a; Zhang et al., 2008). The resulting synthesized relaxin family analogs had their B-chains functionalized with a propargylglycine containing an alkyne group at the N-terminus (Figure 1).

### Click chemistry: attachment of cy5.5 fluorophore to relaxin family analogs

Cy5.5 fluorophore bearing an azide moiety (Lumiprobe, Hallendale Beach, FL, USA) was coupled to alkyne-functionalized H2 relaxin, H2:A(4-24)(F23A) or INSL3 peptides via click chemistry. The alkyne peptide (50 μg) was firstly dissolved in 2 M triethylammonium acetate buffer, pH 7. Next, 2 equivalents of Cy5.5 azide dissolved in dimethyl sulfoxide (DMSO) was added and the reaction was left for 10 min at room temperature. The addition of 21 equivalents of copper (II) sulfate pentahydrate dissolved in dH_2_O and 21 equivalents of ascorbic acid dissolved in dH_2_O were added to the reaction mixture and stirred for an hour at room temperature. The reaction mixture was then diluted with 80 μl of dH_2_O before being injected onto RP-HPLC for purification.

### Peptide characterization

Peptides were purified using RP-HPLC via a preparative column while the final purity of individual synthetic peptides was assessed by analytical RP-HPLC using a Vydac C18 column (250 × 4.6 mm, 300Å, 5 μm) with a buffer system of 0.1% trifluoroacetic acid in dH_2_O (buffer A) and 0.1% trifluoroacetic acid in acetonitrile (buffer B). The molecular masses of all analogs were determined by MALDI-TOF mass spectrometry using a Bruker AutoflexII instrument in the linear mode at 19.5 kV. Furthermore, the peptide content for each analog was quantified by amino acid analysis using vapor-phase acid hydrolysis in 6 M hydrochloric acid containing 2% phenol at 110°C over 24 h. The hydrolysate was then converted to stable, fluorescent derivatives using a Waters AccQTag kit. The derivitized amino acids were separated using a Shim-Pak XR ODS column (3 × 75 mm, 2.2 μm) on a Shimadzu microbore RP-HPLC system.

### Ligand binding assay

Human embryonic kidney (HEK-293T) cells stably transfected with RXFP1 or RXFP2 were cultured in RPMI 1640 medium supplemented with 10% fetal calf serum, 100 μg/ml penicillin, 100 μg/ml streptomycin, and 2 mM L-glutamine and plated into 96-well plates pre-coated with poly-L-lysine for whole cell binding assays. Competition binding experiments were conducted with either Eu^3+^-labeled H2 relaxin (Shabanpoor et al., 2012) or Eu^3+^-labeled INSL3 (Shabanpoor et al., 2008) in the absence or presence of increasing concentrations of unlabeled relaxin peptide analogs. Non-specific binding was determined in the presence of an excess of unlabeled peptides (500 nM relaxin or INSL3). Fluorescence measurements were recorded at an excitation wavelength of 340 nm and emission of 614 nm. All data are presented as the mean ± SE of the percentage of total specific binding of triplicate wells, repeated in at least three separate experiments, and curves were fitted using one-site binding curves in GraphPad Prism 4. Statistical differences in pEC_50_ values were analyzed using One-Way analysis of variance (ANOVA) coupled to the Newman Keul's multiple comparison test for multiple group comparisons in GraphPad Prism 4.

### Functional cAMP assay

The ability of the Cy5.5-labeled insulin/relaxin family peptide analogs to stimulate cAMP production was evaluated using a cAMP reporter gene assay as described (Scott et al., 2006). HEK-293T cells co-transfected with either RXFP1 or RXFP2 and a pCRE β-galactosidase reporter plasmid were plated in 96-well plates (Chen et al., 1995). After 24 h, the co-transfected cells were incubated with increasing concentrations of relaxin analogs in parallel to 10 nM of H2 relaxin or INSL3 for RXFP1- or RXFP2- transfected cells, respectively. The amount of cAMP-driven β-galactosidase expression in each well was assessed with a colormetric assay measuring absorbance at 570 nm on a microplate spectrophotometer. Ligand-induced cAMP stimulation was expressed as a percentage of maximal response of H2 relaxin or INSL3 for RXFP1 and RXFP2 cells, respectively. Each data point was measured in triplicate and each experiment conducted independently at least three separate times. Statistical differences in pEC_50_ values were analyzed using One-Way ANOVA coupled to the Newman Keul's multiple comparison test for multiple group comparisons in GraphPad Prism 4.

### Stereotaxic implantation of guide cannula for central administration of peptides

Adult male C57B/6J mice (10-weeks old) were deeply anesthetized by inhalation of 4% isoflurane and anaesthesia was maintained with 2% isoflurane administered at 0.2 l/min through a nasal cone. Mice were secured in a stereotaxic frame and an incision made to expose the skull, which was cleaned with 6% hydrogen peroxide. Two 1 mm-diameter holes were drilled in the skull on either side of the sagittal midline, 3 mm posterior and 3 mm lateral to bregma, to allow small screws (3 mm long) to be secured in order to anchor the guide cannula in place. A third hole of 1 mm in diameter was drilled through the skull to allow implantation of a stainless-steel guide cannula (11 mm long, 24 gauge) just above the lateral ventricle at the coordinates relative to bregma: anterior-posterior −0.46 mm; medial-lateral −0.8 mm; dorsal-ventral −1.8 mm. The guide cannula and screws were fixed in place by application of self-curing acrylic dental cement (Vertex-Dental, the Netherlands).

### Intracerebroventricular infusion of relaxin family peptides

After 5–10 days recovery post-surgery, mice were gently restrained and an injector was inserted through the guide cannula which protruded 0.5 mm into the lateral ventricle. A single 4 μg dose of each peptide (*n* = 3–4) was delivered in 4 μ l of artificial CSF (aCSF; 147 mM NaCl, 4 mM KCl, 0.85 mM MgCl_2_, and 2.3 mM CaCl_2_), over 1 min via a 10 μ l syringe (0.46 mm diameter) mounted on an infusion pump connected to the injector via polyethylene tubing. Following infusion, the injector was left in place for a further 15 s to prevent backflow of the infused peptide up the guide cannula.

### Behavioral testing and brain processing

Following peptide infusion, mice were placed back in their clear-walled home cages for 30 min with access to water (and food) and filmed through the side of the cage for subsequent assessment of behavior. After 30 min, mice were killed by isoflurane inhalation overdose, decapitated, and brains were removed and “post-fixed” by immersion in chilled 4% PFA solution. After fixation at 4°C for 48 h, brains were then transferred to a 20% sucrose solution for cryoprotection at 4°C overnight. Brains were then coated with OCT embedding medium, frozen on dry ice and stored at −80°C. Coronal sections (40 μm) were cut on a cryostat at −19°C through the rostrocaudal brain axis, slide-mounted and coverslipped with fluorescent mounting media. Sections were viewed and images collected using a confocal laser scanning microscope, using laser light at λ of 633 nm, while light between λ of 650–750 nm was detected.

## Results and discussion

**Figure 2:**
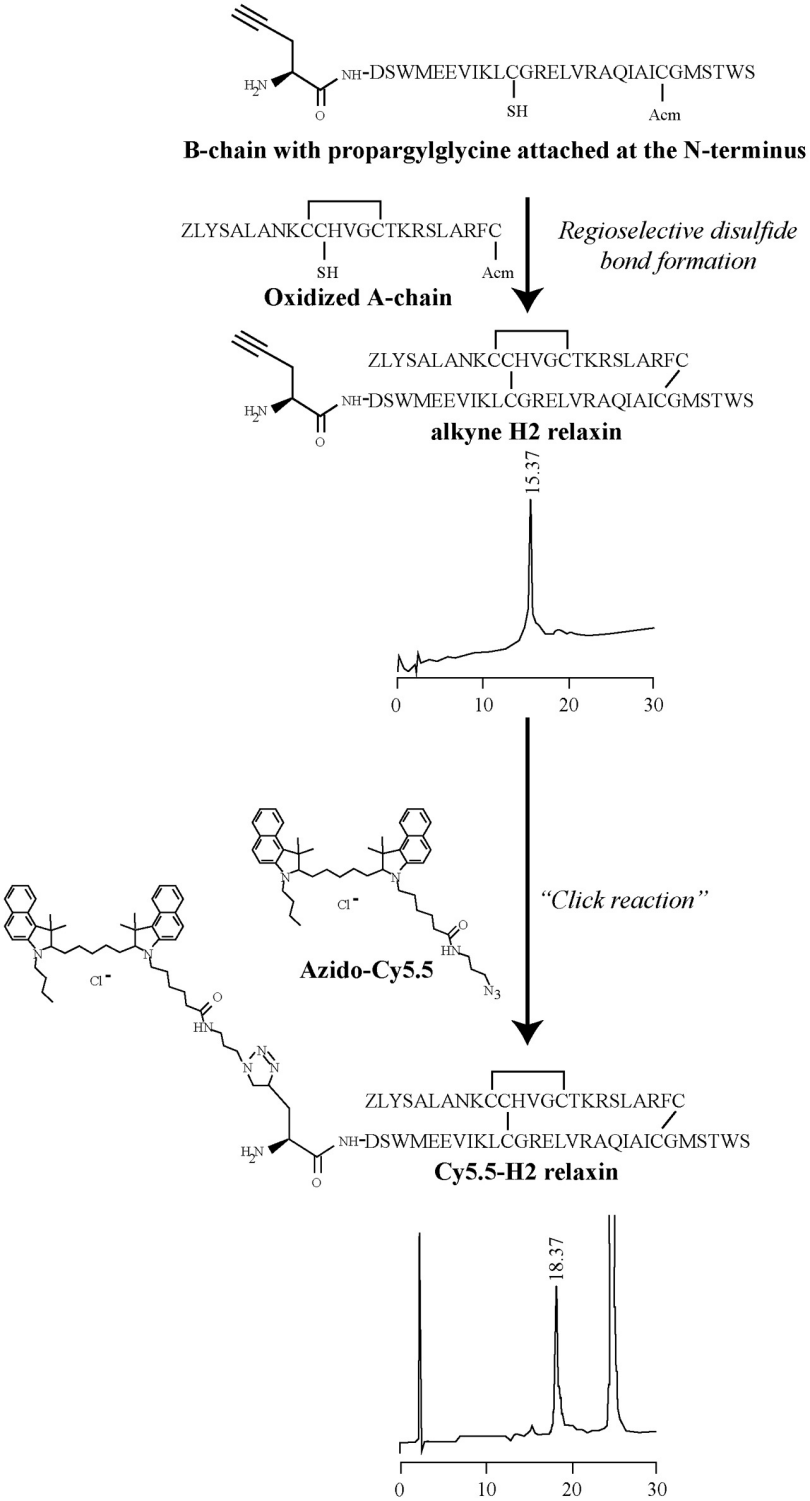
**General schematic representation of the formation of the Cy5.5 analogs**. In step 1, an Fmoc-L-propargylglycine residue with an alkyne moiety was attached at the N-terminus of the B-chain on solid phase and then cleaved from the resin. In step 2, the propargylglycine B-chain was combined with the A-chain, together with the formation of the interdisulfide bonds to give the alkyne product. In step 3, under a copper-catalyzed reaction, the formation of a stable triazole bond between an alkyne and azide group was able to proceed, leading to the formation of the Cy5.5 product of interest. Analytical RPLC was undertaken on a Vydac C18 column (250 × 4.6 mm, 300Å, 5 μm) with a buffer system of 0.1% trifluoroacetic acid in water (buffer A) and 0.1% trifluoroacetic acid in acetonitrile (buffer B). Elution was with a gradient of 20–50% B over 30 min.

The A- and B-chains of H2 relaxin, H2:A(4-24)(F23A), and INSL3 peptides (Figure 1) were synthesized on solid support using Fmoc-SPPS chemistry (Atherton and Sheppard, 1998). An alkyne residue (L-propargylglycine) was coupled to the native B-chain sequences at the N-terminus (Figure 2). Our established regioselective disulfide formation chemical approach was utilized to assemble these two-chain peptides with three differential cysteine S-protecting groups (Trt, tBu, and Acm) used to assist the direct formation of the three disulfide bonds (Bathgate et al., 2006; Hossain et al., 2008a; Zhang et al., 2008). Each peptide was obtained in good overall yields (10–14% relative to starting B-chain material) and was subjected to comprehensive characterization by RP-HPLC and MALDI-TOF mass spectrometry to confirm its high purity and correct molecular mass (Table 1).

**Table 1:** **Competition binding (pKi) and activation (pEC_50_) by H2 relaxin analogs of RXFP1, and by INSL3 analogs of RXFP2, and molecular weight of peptides**.

**Peptide**	**RXFP1**		**RXFP2**		**MW**	
	**pKi**	**pEC_50_**	**pKi**	**pEC_50_**	**Calculated**	**Observed**
Native H2 relaxin	9.45 ± 0.14	10.71 ± 0.14	ND	ND	5961.23	5960.33
Cy5.5-H2 relaxin	7.79 ± 0.26	8.99 ± 0.23	ND	ND	6781.25	6780.81
Cy5.5-H2:A(4-24)(F23A)	7.63 ± 0.25	8.61 ± 0.06	NA	NA	6301.61	6301.68
Native INSL3	NA	NA	9.09 ± 0.22	10.40 ± 0.07	6778.54	6279.46
Cy5.5-INSL3	NA	NA	8.21 ± 0.28	9.01 ± 0.22	7100.38	7100.15

Three peptides [H2 relaxin, H2:A(4-24)(F23A) and INSL3] were successfully labeled in solution with Cy5.5, using the versatile method of click chemistry (Figure 2) (Best, 2009). In this study, the click reaction was used for bioconjugation to enable the labeling of alkyne-functionalized relaxin family peptides with Cy5.5, a fluorescent tag bearing an azide moiety. The Cy5.5 fluorophore was chosen due to its favorable properties. Specifically, it emits in the far red region of the electromagnetic spectrum (excitation maximum, λ^ex^_max_ = 673 nm; Cy5.5 emission maximum, λ^fluor^_max_ = 707 nm) and provides a high signal to noise ratio in tissue studies, as biological specimens and tissues usually have low autofluorescence in this spectral region. The Cy5.5 fluorophore is also compatible for use with more commonly used fluorophores that emit in the blue, green and red spectra, thus allowing for easy labeling and localization of two or more labels in cells and tissues. Our previous studies revealed that attachment of biotin to the N-terminus of the H2 relaxin B-chain via an amide bond resulted in a synthetic H2 relaxin analog able to retain near-native activity (Mathieu et al., 2001). This result indicated that the bulky biotin molecule was far enough away from the known peptide active site region, to avoid any marked effect on its receptor binding and activation properties. Therefore, the Cy5.5 fluorophore was click conjugated to the N-terminus of the B-chain of the peptides. The alkyne moiety of native H2 relaxin, H2:A(4-24)(F23A) and INSL3 reacted with the azide moiety of the Cy5.5 fluorophore forming a triazole ring. Comprehensive characterization by RP-HPLC and MALDI-TOF mass spectrometry confirmed the expected purities and identities (Table 1). Importantly, the data provided no evidence that the conjugation conditions caused a reduction of any of the disulfide bonds or other deleterious side reactions. To our knowledge, this is the first report of a successful copper (II)-mediated Huisgen cycloaddition in the presence of preformed disulfide bonds.

**Figure 3:**
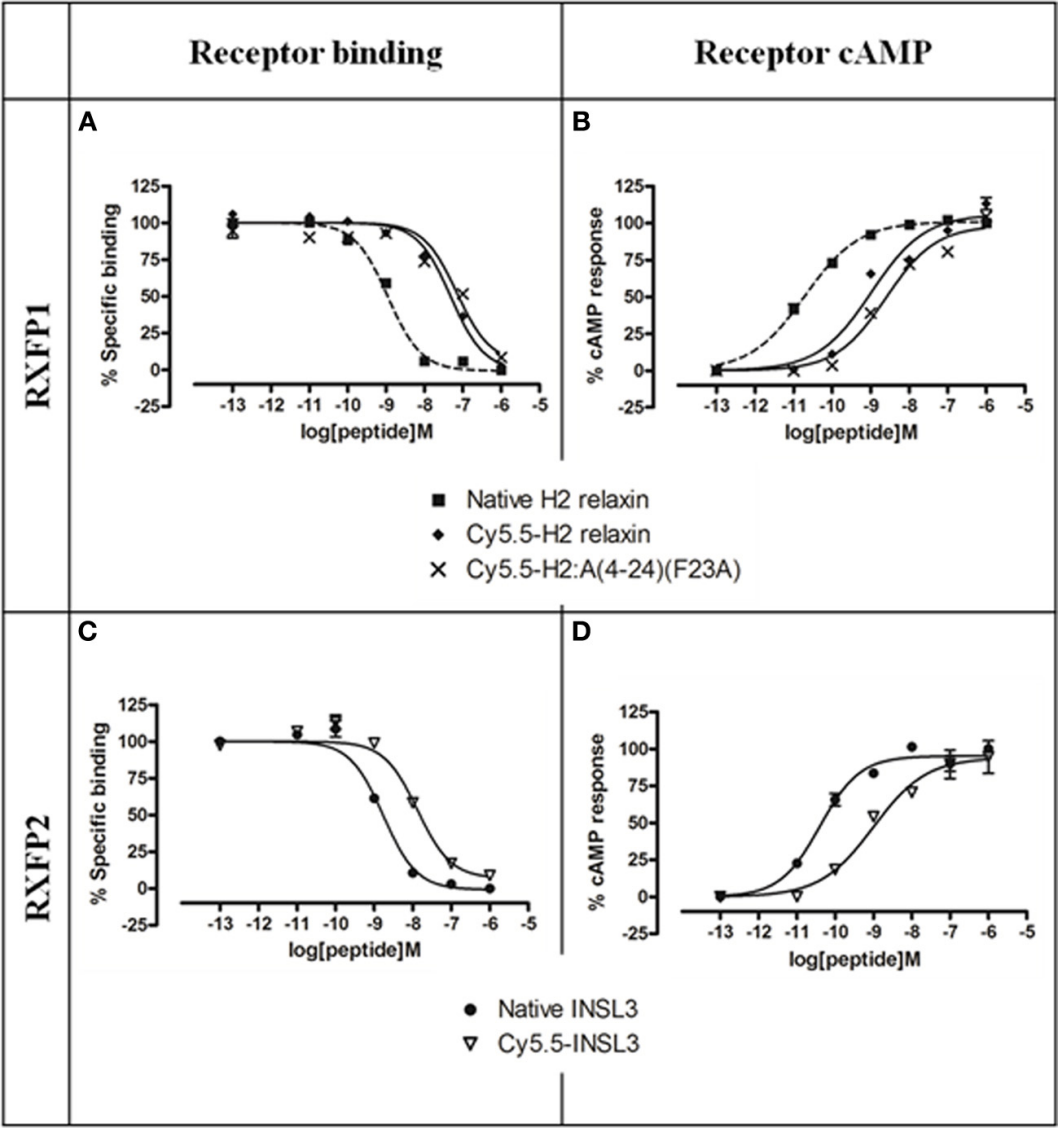
**Activity of Cy5.5 insulin/relaxin family peptide analogs at RXFP1 or RXFP2. (A)** Competition binding of native relaxin and Cy5.5-H2 relaxin analogs in the presence of the competitive ligand Eu^3+^-labeled H2 relaxin tested in HEK-293T cells stably expressing RXFP1. **(B)** Effect of native H2 relaxin and Cy5.5-H2 relaxin analogs on cAMP-related activity in HEK-293T cells expressing RXFP1 using a pCRE-galactosidase reporter gene system. **(C)** Competition binding of native INSL3 and Cy5.5-INSL3 in the presence of the competitive ligand Eu^3+^-labeled INSL3 tested in HEK-293T cells stably expressing RXFP2. **(D)** Effect of native INSL3 and Cy5.5-INSL3 on cAMP activity in HEK-293T cells expressing RXFP2 using a pCRE-galactosidase reporter gene system. Data are expressed as a percentage of specific binding or maximum relaxin/INSL3-stimulated cAMP response and are pooled data from at least three experiments performed in triplicate.

Established cell-based assays were used to evaluate the interaction of the fluorescent analogs at RXFP1 and RXFP2. Both native H2 relaxin and the labeled Cy5.5-H2 relaxin bound to and activated both RXFP1 and RXFP2 (Figure 3). As predicted, the H2 relaxin analog, Cy5.5-H2:A(4-24)(F23A), selectively bound to and activated RXFP1 (Figures 3A,B). In contrast, INSL3 and its labeled analog, Cy5.5-INSL3, only bound to and activated RXFP2 (Figures 3C,D). Notably, due to the addition of the Cy5.5 fluorophore at the N-terminus of the B-chain, the affinity/potency of all labeled analogs was not markedly reduced relative to the native peptides (Figure 3 and Table 1).

It is well established that central activation of RXFP1 (specifically, in the circumventricular organs) confers a dipsogenic response in rodents comparable in magnitude to that elicited by angiotensin II (Harland et al., 1988; Sunn et al., 2002). Icv infusion of Cy5.5-H2 relaxin and Cy5.5-H2:A(4-24)(F23A) stimulated a similar increase in drinking behavior which occurred between 10 and 15 s after mice were placed back into their cages, confirming the *in vivo* biological activity of these peptides (see video in Supplementary Material). In contrast, icv infusion of Cy5.5-INSL3 did not initiate a drinking response. These data confirm a predicted aspect of *in vivo* specificity of Cy5.5-INSL3, i.e., a lack of cross reactivity with RXFP1. But once a clear, reproducible behavioral response in mice to RXFP2 activation has been established, it would be important to determine whether icv injection of Cy5.5-INSL3 confers a similar response (see also below).

**Figure 4:**
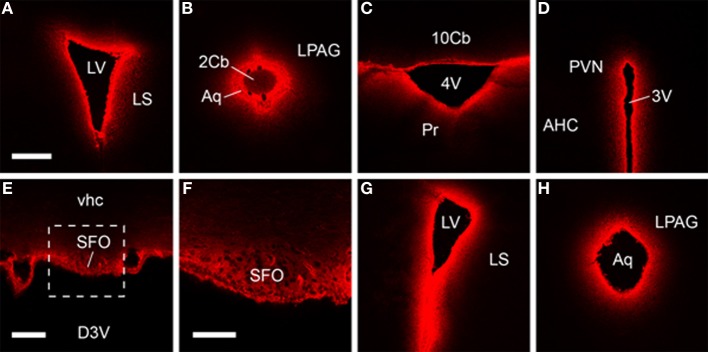
**Fluorescent confocal micrographs of coronal sections from brains harvested 30 min after icv infusion, which demonstrate Cy5.5-H2 relaxin (A–F), Cy5.5-H2:A(4-24)(F23A) (G) and Cy5.5-INSL3 (H) highly concentrated within brain tissue proximal to the cerebral ventricle wall**. Rostral-dorsal location, relative to bregma (mm): **(A)** 0.38; **(B)** −4.96; **(C)** −6.24; **(D)** −0.82; **(E,F)** −0.46; **(G)** 0.86; **(H)** −4.84. Scale bars, **(A–D,G,H)** 300 μm; **(E)** 100 μm; **(F)** 50 μm. The region in **(E)** designated by the square is magnified in **(F)**. Abbreviations: 2/10cb, 2nd, and 10th cerebellar lobules; 3V/4V, 3rd and 4th ventricle; AHC, anterior hypothalamic area, central part; Aq, aqueduct; D3V, dorsal 3rd ventricle; LPAG, lateral periaqueductal gray; LS, lateral septum; LV, lateral ventricle; Pr, prepositus nucleus; PVN, paraventricular hypothalamic nucleus; SFO, subfornical organ; vhc, ventral hippocampal commissure.

Subsequent histological visualization revealed that 30 min after icv injection of Cy5.5-H2 relaxin, high levels of Cy5.5 were present within the ventricle wall and brain tissue proximal to the major cerebral ventricles, including the subfornical organ (Figures 4A–F). At this time point, a diminishing concentration gradient was evident, with brain tissue further than 300 μm from the ventricle wall displaying little or no Cy5.5 fluorescence that was clearly distinguishable from background fluorescence and/or that displayed a clear correlation with the known distribution of RXFP1. Indeed, a similar “peri-ventricular” distribution pattern and relative signal strength was observed following icv infusion of Cy5.5-H2:A(4-24)(F23A) and Cy5.5-INSL3 (Figures 4G,H). Although it is possible that addition of the Cy5.5 fluorophore may impede the diffusion of these peptides within the brain, such effects are likely to be minimal based on the relatively small size of Cy5.5, compared to the three relaxin family analogs (*ca*. 6000 Da). Hence the observed distribution of the Cy5.5 fluorescence should be representative of the distribution of the labeled and the equivalent unlabeled peptides.

These data and earlier studies suggest, however, that although peptides are transported rapidly through the cerebrospinal fluid following icv infusion, all brain structures are not uniformly reached by relaxin family analogs, and hence analysis of any behavioral responses should be interpreted accordingly. For example, *in situ* hybridization (Piccenna et al., 2005) and radioligand binding studies (Ma et al., 2006) have revealed that RXFP1 mRNA/binding sites are broadly distributed within the rodent brain including high densities in the olfactory bulb, cortex, hippocampus and subiculum, amygdala, circumventricular organs, thalamus, hypothalamus, and brainstem (Ma and Gundlach, 2007; Gundlach et al., 2009). This broad distribution suggests that relaxin/RXFP1 signaling modulates a diverse range of neural circuits, although the nature of this modulation and the relative role of endogenously/locally produced relaxin vs. any effects of circulating relaxin are not known. In fact, studies of the effects of icv infusion of H2 relaxin have thus far linked central RXFP1 activation with relatively few behavioral responses [e.g., (Summerlee et al., 1998; McGowan et al., 2010)], while local infusion of H2 relaxin into a deeper area of the rat brain, the basolateral amygdala, produced effects on fear memory (Ma et al., 2005). Notably, the diffusion and distribution of the H2 relaxin was not monitored in the former icv studies, whereas injection of H2 relaxin into an adjacent area of the amygdala (central nucleus) was without effect in the latter study.

Similarly, although INSL3/RXFP2 signaling is best characterized for its roles in peripheral tissues and in reproduction (Bogatcheva et al., 2003; Kamat et al., 2004; Ferlin et al., 2006; Bathgate et al., 2013), RXFP2 is also broadly expressed within cortical, striatal, thalamic, and hypothalamic areas of rodent brain (Sedaghat et al., 2008; Gundlach et al., 2009). Currently, the effects of central INSL3/RXFP2 activation remain largely unreported, and studies in this area should benefit from the availability of Cy5.5-INSL3.

The approach used here to label H2 relaxin, H2:A(4-24)(F23A) and INSL3 might also be applied to other relaxin family members, as icv infusion of H3 relaxin and analogs modulates feeding via interactions with RXFP3 within the hypothalamus (McGowan et al., 2007; Ganella et al., 2012), while local infusions of RXFP3 agonist or antagonist peptides into the medial septum alters spatial memory (Ma et al., 2009). Overall, these studies indicate that behaviors altered following icv infusion of relaxin family peptides (and other peptides) may represent actions mediated by activated or inhibited receptor populations proximal to the main cerebral ventricular system.

However, although a bias may exist for the modulation of receptors near the ventricular wall, it is possible that receptors in deeper brain structures are occupied by peptides administered icv. In a recent study that addressed this issue directly using a similar protocol and time frame, fluorophore-conjugated neuropeptide S (NPS) was observed bound to NPS receptor-positive neurons in deep brain areas at levels well above that of the surrounding tissue after icv and intranasal administration (Ionescu et al., 2012). Similar studies could be conducted using fluorophore-conjugated relaxin family analogs. If the dose, route of administration and timing of the analysis can be optimized, receptor bound peptides may be identified bound to, or within, RXFP1 or RXFP2-expressing neurons. Clearly these studies would benefit from an ability to “label” RXFP1- or RXFP2-positive neurons using either immunohistochemistry or transgenic reporter mouse strains, as currently detection of Cy5.5 associated with individual neurons (even in the subfornical organ) is difficult. If successful, however, these studies might provide insights into whether H2 relaxin or INSL3 is internalized after binding to RXFP1 and RXFP2 *in vivo*, as *in vitro* studies suggest that this is not the case (Callander et al., 2009). Cy5.5-peptides could also help determine whether the changes in neuronal activity observed following icv infusion of H2-relaxin (via Fos immunostaining) (McKinley et al., 1997) are the result of direct or indirect RXFP1 (or RXFP2) signaling.

Further studies may also benefit from utilizing different methods of tissue processing to the paraformaldehyde “post-fixation” technique used here. Although this method allowed a “snap shot” visualization of the peptide distribution, the vast majority of fluorophore/peptide fixed within the brain tissue was not enriched on/within neurons—a pattern which, if observed, might have reflected binding to RXFP1 or RXFP2. Indeed, the presence of covalently cross-linked peptide fixed within surrounding tissue may provide unwanted background signal that hampers the visualization of peptides specifically bound to neurons, particularly in periventricular regions such as the subfornical organ where such background signal was high. It is possible that the level of non-specifically bound peptides could be reduced by a brief (optimized) perfusion of the brain with buffered saline prior to removal, followed by freezing, and subsequent post-fixing of slide-mounted sections.

In addition to studies employing the icv route of administration, tracking the degree of peptide diffusion/spread, and confirming the correct location of an injection in a target area is also important for studies of local peptide injections. Cy5.5-peptides can also be used in combination with real-time whole animal fluorescent imagers, to determine the rate at which relaxin family analogs cross the blood brain barrier and enter the brain following systemic infusion.

## Conclusion

These studies demonstrate the utility of synthetic chemistry to generate fluorophore-conjugated relaxin family analogs that retain activity and good receptor selectivity *in vitro* and *in vivo*. The fluorophore can be used to visualize the location/distribution of these peptides in biological tissues, which avoids the disadvantages of using radiolabeled analogs with their associated safety hazard and possible short half-life. Here we observed that Cy5.5-conjugated relaxin family peptides administrated icv into mice displayed an appropriate profile of acute biological activity and at 30 min post-infusion accumulated within brain regions proximal to the cerebral ventricular system; however, further optimized studies are required to visualize specific receptor occupation on/within neurons. In summary, we describe the generation of novel chemical tools to further probe the function of H2 relaxin/RXFP1 and INSL3/RXFP2 signaling that offer characteristics which can assist in the interpretation of pharmacological studies both *in vitro* and *in vivo*.

## Author contributions

Mohammed Akhter Hossain and John D. Wade conceived the project, and designed and coordinated the research. Linda J. Chan chemically synthesized and analyzed the relaxin family peptides, and performed *in vitro* assays. Craig M. Smith and Berenice E. Chua produced the *in vivo* data. Feng Lin provided essential reagents and undertook the amino acid analyses. Ross A. D. Bathgate provided bioassay data. Linda J. Chan, Craig M. Smith, Ross A. D. Bathgate, Frances Separovic, Andrew L. Gundlach, Mohammed Akhter Hossain, and John D. Wade analyzed and compiled the data and/or co-wrote the manuscript. The final manuscript was read and approved by all the authors.

## Conflict of interest statement

The authors declare that the research was conducted in the absence of any commercial or financial relationships that could be construed as a potential conflict of interest.
